# Seventh BHD international symposium: recent scientific and clinical advancement

**DOI:** 10.18632/oncotarget.28176

**Published:** 2022-01-20

**Authors:** Mark R. Woodford, Avgi Andreou, Masaya Baba, Irma van de Beek, Chiara Di Malta, Iris Glykofridis, Hannah Grimes, Elizabeth P. Henske, Othon Iliopoulos, Masatoshi Kurihara, Romain Lazor, W. Marston Linehan, Kenki Matsumoto, Stefan J. Marciniak, Yukiko Namba, Arnim Pause, Neil Rajan, Anindita Ray, Laura S. Schmidt, Wei Shi, Ortrud K. Steinlein, Julia Thierauf, Roberto Zoncu, Anna Webb, Mehdi Mollapour

**Affiliations:** ^1^Department of Urology, SUNY Upstate Medical University, Syracuse, NY, USA; ^2^Department of Biochemistry and Molecular Biology, SUNY Upstate Medical University, Syracuse, NY, USA; ^3^Upstate Cancer Center, SUNY Upstate Medical University, Syracuse, NY, USA; ^4^Department of Medical Genetics, School of Clinical Medicine, University of Cambridge, Cambridge, UK; ^5^International Research Center for Medical Sciences (IRCMS), Kumamoto University, Kumamoto, Japan; ^6^Department of Human Genetics, Amsterdam UMC, Vrije Universiteit Amsterdam, Amsterdam, The Netherlands; ^7^Telethon Institute of Genetics and Medicine (TIGEM), Naples, Italy; ^8^Medical Genetics Unit, Department of Medical and Translational Science, Federico II University, Naples, Italy; ^9^Amsterdam UMC, Location VUmc, Human Genetics Department, Cancer Center Amsterdam, Vrije Universiteit Amsterdam, Amsterdam, The Netherlands; ^10^Cambridge Institute for Medical Research, Cambridge Biomedical Campus, University of Cambridge, Cambridge, UK; ^11^Center for LAM Research and Clinical Care, Brigham and Women’s Hospital, Harvard Medical School, Boston, MA, USA; ^12^Center for Cancer Research, Massachusetts General Hospital Cancer Center, Harvard Medical School, Boston, MA, USA; ^13^Division of Hematology-Oncology, Department of Medicine, Massachusetts General Hospital, Boston, MA, USA; ^14^Pneumothorax Research Center and Division of Thoracic Surgery, Nissan Tamagawa Hospital, Setagayaku, Tokyo, Japan; ^15^Respiratory Medicine Department, Lausanne University Hospital and University of Lausanne, Lausanne, Switzerland; ^16^Urologic Oncology Branch, Center for Cancer Research, National Cancer Institute, Bethesda, MD, USA; ^17^Department of Respiratory Medicine, Addenbrooke's Hospital, University of Cambridge, Cambridge, UK; ^18^Division of Respiratory Medicine, Juntendo University Graduate School of Medicine, Tokyo, Japan; ^19^Department of Biochemistry, Goodman Cancer Research Institute, McGill University, Montréal, Canada; ^20^Translational and Clinical Research Institute, Newcastle University, Newcastle upon Tyne, UK; ^21^Indian Statistical Institute, Kolkata, WB, India; ^22^Basic Science Program, Frederick National Laboratory for Cancer Research, Frederick, MD, USA; ^23^The Saban Research Institute, Children's Hospital Los Angeles, The Keck School of Medicine, University of Southern California, Los Angeles, CA, USA; ^24^Institute of Human Genetics, University Hospital, Ludwig Maximilian University (LMU) Munich, Munich, Germany; ^25^Department of Pathology, Center for Integrated Diagnostics, Massachusetts General Hospital, Harvard Medical School, Boston, MA, USA; ^26^Department of Otorhinolaryngology, Head and Neck Surgery, Heidelberg University Hospital and Research Group Molecular Mechanisms of Head and Neck Tumors, German Cancer Research Center (DKFZ), Heidelberg, Germany; ^27^Department of Molecular and Cell Biology, University of California at Berkeley, Berkeley, CA, USA; ^28^The BHD Foundation, The Myrovlytis Trust, London, UK

**Keywords:** Birt-Hogg-Dubé syndrome, folliculin, FLCN, tuberous sclerosis complex, LDHA

## Abstract

The 7th Birt-Hogg-Dubé (BHD) International Symposium convened virtually in October 2021. The meeting attracted more than 200 participants internationally and highlighted recent findings in a variety of areas, including genetic insight and molecular understanding of BHD syndrome, structure and function of the tumor suppressor Folliculin (FLCN), therapeutic and clinical advances as well as patients’ experiences living with this malady.

## INTRODUCTION

**Figure 1:**
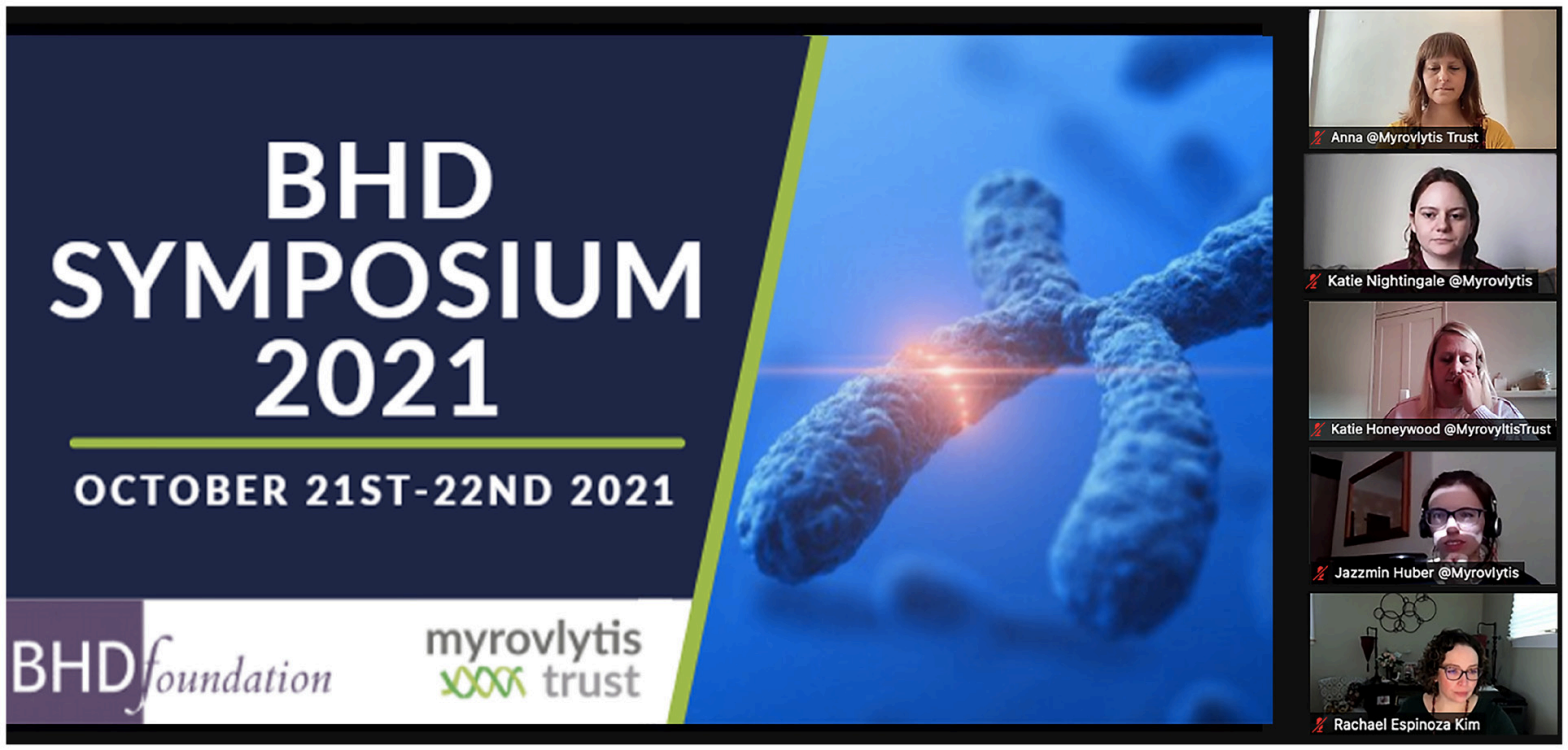
BHD international symposium 2021 was held with great success on October 21–22, 2021 with over 200 participants including scientists, clinicians and patients. We thank members of BHD foundation and Myrovlytis Trust team for creating, organizing and maintaining an inspiring and inclusive environment for research investigators to share their findings for the benefit of BHD patients.

The last in-person BHD symposium was organized by Dr Gennady Bratslavsky & Dr Mehdi Mollapour (SUNY Upstate Medical University, USA) and held in Syracuse, NY, USA in 2015. Additionally, the ongoing COVID-19 pandemic has disrupted the ability of the scientific community to organize and host conferences in person. Despite these obstacles, the team at the BHD foundation & Myrovlytis Trust, including Dr Anna Webb (Director), Dr Jazzmin Huber and Dr Katie Nightingale (Charity Officers) and Ms Katie Honeywood (Office Manager) organized the virtual meeting with help from the organizing committee. More than 200 BHD patients, leading researchers and surgeons from across the globe were brought together to exchange their data and findings to understand this disease and identify different strategies to treat patients with BHD syndrome (Figure 1).

### Birt-Hogg-Dubé (BHD) syndrome

Birt-Hogg-Dubé (BHD) syndrome is a rare inherited condition that predisposes affected individuals to develop spontaneous pneumothorax, pulmonary cysts, and benign skin tumors (fibrofolliculomas) as well as renal tumors [1]. These kidney masses present with diverse histology, ranging from chromophobe renal cell carcinoma (RCC) to benign oncocytoma, as well as hybrid tumors containing features of both chromophobe RCC and oncocytomas [2–4].

In the opening keynote talk, Dr. W. Marston Linehan, Chief of the Urologic Oncology Branch at the National Cancer Institute (NCI), National Institute Health (NIH), described familial RCC as a part of BHD [5, 6]. He further showed how work by his lab on genetic linkage analysis led to identification of *FLCN* as the BHD gene [7], finding *FLCN* pathogenic mutations in 96% of BHD families. Dr Linehan further demonstrated the development of a surgical management approach for patients with BHD-associated renal cell carcinoma that involves active surveillance until the largest renal tumor reaches the 3 cm threshold [4, 8]. In order to provide the foundation for the development of a therapeutic approach for treatment of BHD patients, his group has studied the *FLCN* gene pathway and identified FLCN-interacting binding partners, co-chaperones FNIP1 and FNIP2, and shown that FLCN regulates activation and nuclear translocation of TFE3 [9–19].

### Genetic insight into BHD

Unlike other international meetings where leaders in the field follow the keynote speaker, in this meeting students, post-doctoral fellows and junior principal investigators were given the opportunity to share their data. Dr Yukiko Namba (The Juntendo University Graduate School of Medicine, Japan) presented data on the clinical features of 335 Asian patients with BHD-associated lung symptoms. In this cohort, skin lesions were found in 34% and renal tumors in 4% of patients. Approximately 25% of patients developed the first pneumothorax episode prior to the age of 25 and onset age was younger in male than female patients. Clinical course after the establishment of diagnosis remained unknown, therefore a registry system is needed to disclose the comprehensive clinical pictures of BHD [20, 21].

One of the outstanding questions in the field is the prevalence of BHD in the general population. Dr Romain Lazor (Lausanne University Hospital and University of Lausanne, Switzerland) applied the Bayes theorem of conditional probability to epidemiological data on spontaneous pneumothorax and showed that the prevalence of BHD in the general population is about 2 cases per million, without difference between genders [22]. Dr Anindita Ray from Dr Bidyut Roy’s group (Indian Statistical Institute, India) presented the results of the first comprehensive genetic study in 31 BHD-patients with spontaneous pneumothorax or pulmonary cysts and their 74 asymptomatic family members from 15 families in India. They sequenced the *FLCN* gene and found variants in genes associated with homocystinuria for some patients with BHD presenting features. However, they did not find pathogenic mutations in 9 clinically diagnosed patients (29%), which perhaps indicates an undescribed gene association and/or a larger mutational spectrum in the Indian or South-Asian population.

The initial findings from the 100,000 Genomes Project analysis of Familial Pneumothorax in the UK suggest that clinically, cases of known pneumothorax syndromes are being efficiently identified and they are considered as differential diagnoses. Dr Hannah Grimmes (University of Cambridge, UK) from Dr Marciniak’s lab demonstrated that only one patient from a cohort of 33 patients represented a missed diagnosis of BHD. Analysis of the remaining 32 patients’ genomes has generated multiple candidate genes that could be involved in familial pneumothorax. The preliminary results of this analysis suggest that familial pneumothorax may be a collection of ultra-rare disorders. Dr Avgi Andreou (University of Cambridge, UK) from Dr Eamonn R Maher’s group presented their analysis performed with Dr Bryndis Yngvadottir on the frequency of pathogenic germline variants in cancer susceptibility genes (CSGs) in 1,336 participants with renal cell carcinoma RCC. Approximately 6% of patients with RCC unselected for family history, early onset of RCC or presence of features associated with an inherited RCC syndrome have a pathogenic germline variant in a cancer susceptibility gene (CSG), with 1 in 300 having a pathogenic germline *FLCN* variant. Dr Kenki Matsumoto (University of Cambridge, UK) presented his work on investigating the existence of pneumothorax-only pathogenic variants (POPVs) in BHD patients. His systematic literature review did not find any difference in variant type or location between POPVs and all other variants [23]. The existence of POPVs in the literature is likely to be as a result of confounding factors such as age of individuals and number of individuals affected by a particular variant. His findings provide a recommendation for all BHD patients to have renal cell carcinoma screening regardless of the underlying FLCN variant.

### Molecular understanding of BHD syndrome

#### Cross-talk between Birt-Hogg-Dubé and Tuberous Sclerosis Complex (TSC) syndromes

Individuals with tuberous sclerosis complex (TSC) and BHD develop cystic lung disease with pneumothorax and renal cell carcinoma (RCC). The associated RCC subtypes have distinctive histologic features, including chromophobe and hybrid oncocytic-chromophobe tumors (HOCT) [24], however the molecular pathways underlying these overlapping clinical features are poorly understood. Dr Lisa Henske (Brigham and Women’s Hospital, Harvard Medical School, USA) presented their work on upregulation of lysosomes and lysosomal genes in TSC1-deficient and TSC2-deficient cells. The transcription factors TFEB and TFE3 are unexpectedly localized to the nucleus in TSC1-deficient and TSC2-deficient cells and in FLCN-deficient cells [25], where they regulate lysosomal biogenesis and cell proliferation. In agreement, the Ballabio group has recently shown that TFEB is responsible for renal cyst formation in a mouse model of BHD [27]. These data suggest that TFEB and TFE3 may be the “missing links” between the pulmonary and renal phenotypes of TSC and BHD, via lysosomal biogenesis and lysosomal exocytosis.

Dr Chiara Di Malta (TIGEM & Federico II University, Italy) presented her work in collaboration with Dr Andrea Ballabio on the role of MiT/TFE transcription factors, in particular TFEB, in BHD syndrome. She found that mTORC1-mediated inhibition of TFEB is strictly dependent on the GAP activity of FLCN. Therefore, loss of FLCN in BHD results in constitutive activation of TFEB which, in turn, promotes mTORC1 hyper-activation by increasing the levels of the Rag GTPases [26]. Importantly, depletion of TFEB completely rescued the phenotype of kidney-specific FLCN knock-out mice [27]. Her findings suggest that MiT/TFE factors play a key role in the growth of kidney tumors associated with BHD syndrome.

FLCN is dependent on the interacting proteins FNIP1 and FNIP2 for stability in normal cells [15]. Dr Mark Woodford (SUNY Upstate Medical University, USA) presented data showing that the FLCN-FNIP relationship is mediated by the molecular chaperone Hsp90. FNIP1/2 regulate the chaperone activity of Hsp90, conferring stability to FLCN and other Hsp90-dependent proteins, designating FNIP1/2 as Hsp90 co-chaperones [28]. Disease-associated mutations in FLCN lead to premature truncation of FLCN protein products, precluding interaction with FNIP1/2 [28]. In the case of a patient with BHD syndrome, the tumor suppressor co-chaperone Tsc1 compensated for FNIP1 in chaperoning mutant FLCN protein [29]. Misappropriation of Tsc1 destabilized the Tsc1-dependent tumor suppressor Tsc2 and led to the development of a renal angiomyolipoma, a tumor subtype more commonly associated with Tsc1/2 inactivating mutations [30].

#### FLCN structure and function

Dr Iris Glykofridis (Amsterdam UMC, The Netherlands) from Dr Wolthuis’s lab, presented her work on unique transcriptomes and proteomes of newly generated isogenic cell lines (FLCNneg vs. FLCNpos) and revealed a broad spectrum of biological processes regulated by FLCN. Using Gene Set Enrichment and transcription motif analyses she confirmed earlier observations regarding FLCN-mediated regulation of the TFE transcription factors. Interestingly, her experiments also revealed that FLCN loss induced a non-canonical interferon response signature. She concluded that this FLCN dependent interferon expression signature is induced by activation of STAT1/2 and appears to counterbalance TFE-directed hyper-proliferation in renal cells [31]. The contribution of these processes towards renal tumorigenesis remains unknown.

Dr Arnim Pause (McGill University, Canada) revealed that FLCN, FNIP1 and FNIP2 are downregulated in many human cancers, including invasive basal-like breast carcinomas. In contrast AMPK and TFE3 targets are activated when compared to the less aggressive, luminal subtypes. He further showed that FLCN loss in luminal subtypes promotes tumor growth through TFE3 activation and subsequent induction of glycolysis and angiogenesis, which are controlled by activation of the PGC-1α/HIF-1α pathway. Interestingly, AMPK enhances TFEB/3 transcriptional activity through phosphorylation of C-terminal serine residues, and consequently induces chemoresistance in cells. Thus, FLCN loss appears to induce TFE3-dependent breast tumor growth through activation of multiple mechanisms that reveal a general role of a deregulated FLCN/AMPK/TFE3 pathway in human cancers. AMPK could be as a promising upstream target in cancer therapy to evade chemotherapeutic resistance. Dr Roberto Zoncu (University of California at Berkeley, USA) presented recent structural and functional data on the lysosomal folliculin complex (LFC), consists of a lysosome-localized protein complex that includes FLCN, FNIP2, Rag GTPases and Ragulator. He discussed the putative role for the LFC as a checkpoint that enables mTORC1-dependent phosphorylation of the transcription factor TFEB, and how LFC disruption upon FLCN loss may contribute to the pathogenesis of BHD syndrome. BHD-associated renal cell carcinomas exhibit metabolic deregulation, evidenced by increased activity of lactate dehydrogenase (LDH) and elevated lactate production [32, 33]. Dr Othon Iliopoulos (Massachusetts General Hospital and Harvard Medical School, USA) presented his work showing that activation of the small GTPase Rab7a depends on FLCN GAP activity. Rab7a is involved in endocytic trafficking of EGFR and suppresses EGFR ligand-dependent activation. Mutant FLCN proteins fail to activate Rab7a, promoting EGFR internalization and downstream mTOR activation [34]. These data suggest inhibition of receptor tyrosine kinases may be a viable therapeutic strategy in patients harboring inactivating *FLCN* mutations.

Dr Mehdi Mollapour (SUNY Upstate Medical University) presented new data demonstrating that FLCN functions by directly and specifically inhibiting lactate dehydrogenase isoform A (LDHA). FLCN inhibition of LDHA was lost in cancer cell lines derived from a variety of origin tissues, providing a potential explanation for the widespread observation of metabolic dysregulation in cancer. A decameric peptide derived from FLCN was sufficient to bind and inhibit LDHA in renal cell carcinomas, leading to cell death [35]. These findings [34] provide a *bona fide* function for the FLCN tumor suppressor.

#### Animal models of BHD

Dr Masaya Baba (Kumamoto University, Japan) showed that endothelial cell specific *FLCN* knockout led to misconnection of blood vessels and lymphatic vessels in mice, caused by nuclear localization of *TFE3* followed by ectopic *PROX1* expression in vascular endothelial cells. In addition, he observed dilated lymphatic vessels filled with red blood cells in the lungs of BHD patients, which may be responsible for lung cyst formation in BHD syndrome. Dr Wei Shi (Children’s Hospital Los Angeles, USA) focused on pulmonary phenotypic studies in mice with genetic deletion of *FLCN* in lung mesenchymal cells vs. epithelial cells. His data indicated that lung mesenchymal *FLCN* knockout results in early defective alveolar growth and late lung cystic lesions. Dr Shi’s data also suggested that lung mesenchyme-specific *FLCN* knockout mice may be a better model for studying pulmonary cystic lesions in BHD [36]. Dr Laura Schmidt (NCI, USA) used high-throughput small molecule screens in FLCN-deficient human kidney cancer cell lines to identify several classes of compounds that were cytotoxic *in vitro* and subsequently tested their therapeutic efficacy in the *FLCN*-mutant Nihon rat renal tumor model. Treatment with inhibitors of the PI3K/mTOR pathway significantly reduced tumor growth rates, whereas an inhibitor of the proteasome showed only modest effect depending on tumor size, and inhibitors of histone deacetylases and DNA topoisomerase produced no tumor response in this model. The results of this study suggest that PI3K/mTOR inhibitors might be a potential therapeutic option for BHD kidney cancer.

### Latest clinical advances

Dr Ortrud K. Steinlein (LMU Munich, Germany) showed that the rate of colorectal cancer was moderately but significantly increased (5.1% versus 1.5%, *p*-value 0.0068) based on samples collected from 83 BHD families (256 patients). No specific *FLCN* mutation was associated with colorectal cancer diagnosis. Interestingly, 10% of the BHD families either had members affected by colorectal cancer before the age of 50 years or at least three members affected by colorectal cancer and therefore fulfilled the revised Bethesda criteria for HNPCC (hereditary non-polyposis colon cancer). Other tumor types frequently associated with HNPCC were absent, arguing against a coincidence of BHD and HNPCC. These results suggest that BHD itself might be a risk factor for early onset of colorectal cancer. It therefore seems prudent to suggest colonoscopy screening for patients with BHD syndrome at least ten years earlier than usually recommended [37]. Dr Neil Rajan (Newcastle University, UK) presented data on skin tumors arising in BHD. He highlighted how *in silico* reconstruction of skin tumors revealed different spatial relationships between hair follicles and the bulk of the tumor in fibrofolliculoma and trichodiscoma. In addition, he presented clinical follow up data on electrosurgical interventions and their utility in the ablation of BHD skin tumors. Dr Masatoshi Kurihara (Nissan Tamagawa Hospital, Japan) demonstrated that Total Pleural Covering is effective to prevent recurrent pneumothorax in BHD patients. This method is innovative and will spread to prevent pneumothorax in diffuse cystic lung diseases instead of chemical pleurodesis [38, 39]. Dr Irma van de Beek (Amsterdam UMC, The Netherland) showed that establishing the diagnosis of BHD is important because it allows for renal surveillance in the proband and other relatives with the predisposition for BHD. The goal of renal surveillance is the early detection and treatment of renal cell carcinoma. In clinical practice, many newly diagnosed families with BHD in retrospect have features that could have been a clue for an earlier diagnosis. Therefore, raising awareness among other caregivers could be an important way to identify more families with BHD.

### Researcher lightning session

This session, in which four scientists and physicians from the BHD field explained their research focus to patients was hosted by Dr Julia Thierauf (Massachusetts General Hospital and Harvard Medical School, USA & German Cancer Research Center (DKFZ), Germany) and Dr Katie Nightingale (Charity Officers, Myrovlytis Trust). The first speaker, Dr Laura Schmidt (NCI, USA) focused on the genotype-phenotype correlations in the NCI-BHD cohort. Her interest lies in those patients who are clinically positive for BHD but lack FLCN sequence alterations. Dr Neil Rajan (Newcastle University, UK), focused on the skin aspect of BHD and presented 3D reconstructions of fibrofolliculoma. Dr Lisa Henske (Brigham and Women’s Hospital, USA) focused on the pathogenesis and therapy of BHD and TSC. The final panelist, Dr Stefan Marciniak, (University of Cambridge, UK) whose group studies the cell biology of stress signaling caused by protein misfolding, discussed the genetics of disorders that cause pneumothorax. The panelist presentations were followed by questions from patients regarding sufficient treatment of fibrofolliculoma, a longer discussion about the clinical evidence on risk factors for pneumothorax (flying and diving) and how to create more evidence-based data in order to negotiate with health care insurance especially in the United States. Dr Schmidt mentioned an observational study once conducted at the NIH that evaluated pneumothorax events in patients that were seen at the NIH and traveled via airplane. The analysis did not show correlations between the event of pneumothorax and flying. However, the consensus was, that patients with confirmed pneumothorax or suspicion of pneumothorax should not fly or dive before exclusion of such. Treatment of skin lesions related to BHD remains a cumbersome task and despite constant developments in laser treatments for other skin diseases, the successful treatment of fibrofolliculoma is likely a combination of novel topical compounds and laser resurfacing or surgical removal. In order to create a foundation for broad financial coverage from payors, the group agreed that more clinical trials and suitable preclinical models are needed. During the session, patients expressed their desire to participate in studies and asked about who to contact and how to proceed. The BHD foundation is actively helping to connect patients and scientists. This topic led to a final consensus from the scientific presenters, which was how grateful they were to work in such a well-connected field where patients are eager to support science wherever they could.

### Stories from BHD patients

During this session hosted by Dr Julia Thierauf and Dr Jazzmin Huber, four BHD patients shared stories from the moment of their diagnosis to their daily life with BHD. Mr Jim Laycock, a 56 year-old computer programmer from Canada, got his first BHD symptom when his lung collapsed in 1978, at the age of 13, but he did not receive a diagnosis until 2012. His sister ultimately was able to connect the dots after being diagnosed with a kidney tumor years later. She was the one who came up with the differential diagnosis of BHD after conducting her own research on collapsed lungs and kidney neoplasms. Ms Jenny Marlé-Ballangé, who lives on the west coast of France, is a retired university teacher who manages BHD FRANCE (a charity working closely with the BHD foundation). Their aims are to raise awareness of BHD amongst clinicians such as dermatologists, pneumologists and radiologists, as well as patients in France and other francophone countries. She put a lot of emphasis on raising awareness for BHD and communicating complex scientific and clinical terminologies in layman’s terms. Ms Carolyn Lindgren, a mother of three from Chicago, USA, was diagnosed incorrectly three times before being diagnosed with BHD. Ms Anna Britton-Lewis is a 41 year old school lab tech from UK. She got her first skin bump at the age of 12 and was diagnosed with BHD alongside her father. Although the patients had quite different stories about how they were diagnosed with BHD, they all agreed on the hurdles in correctly diagnosing an orphan disease and finding doctors who are familiar with BHD. Jenny shared her opinion that, despite being a cancer syndrome, BHD is not the most malignant form of disease and that patients usually maintain a high quality of life. A discussion about the option to sort FLCN mutant variants via *in vitro* fertilization revealed the very personal aspect of this decision and a consensus could not be reached.

### Concluding remarks

Significant advances have been made during the past five years towards the tumor suppressor function of *FLCN* and its association with BHD syndrome. Previous work, along with findings presented at this meeting, have concluded that FLCN GAP activity is crucial for its role in the regulation of mTOR activity and that loss of FLCN activates AMPK and promotes a TFE3/TFEB transcriptional program. The contribution of these pathways to the pathogenesis of BHD syndrome remains unresolved. The recent identification of LDHA as an intracellular target of FLCN tumor suppressor activity has established a new paradigm for the study of BHD syndrome that reconciles biochemical and genetic findings in the field.

Critically, research on the molecular underpinnings of BHD syndrome has yet to yield actionable therapeutic interventions [40]. Despite this, small molecule antagonists of AMPK, PI3K/mTOR and LDHA present potential opportunities for clinical benefit. The future direction of basic and clinical research remains a focus on addressing two broad and major gaps in our knowledge: Why do kidney tumors occur in BHD patients? and how to develop a strategy for the treatment of BHD syndrome?
